# Frailty and arteriovenous fistula patency in elderly hemodialysis patients: a retrospective cohort study*

**DOI:** 10.1590/1980-220X-REEUSP-2025-0121en

**Published:** 2025-09-01

**Authors:** Polyana Bezerra Mendonça, Rodrigo Bezerra, Flávio Teles, Emmanuelle Tenório Albuquerque Godoi Berenguer de Barros e Silva, Alexandre Cavalcanti de Holanda, Filipe Carrilho de Aguiar, Carolina Sabadoto Brienze, Audes Diógenes Magalhães Feitosa, Wilson Nadruz, Esdras Marques Lins

**Affiliations:** 1 Universidade Federal de Pernambuco, Recife, PE, Brazil.; 2 Universidade Federal de Alagoas, Maceió, AL, Brazil.; 3 Pronto Socorro Cardiológico Universitário de Pernambuco Prof. Luiz Tavares, Recife, PE, Brazil.; 4 Universidade Estadual de Campinas, Campinas, SP, Brazil.

**Keywords:** Arteriovenous Fistula, Renal Dialysis, Frailty, Constriction, Pathologic, Vascular patency, Fístula Arteriovenosa, Diálise Renal, Fragilidade, Constrição Patológica, Patência Vascular

## Abstract

**Objective:**

To evaluate the correlation between frailty and AVF patency type in elderly patients in HD.

**Methods:**

This retrospective cohort study evaluated 89 patients from April 2019 to June 2022. The following data were analyzed: 1) Patient characteristics according to the Clinical Frailty Scale (CFS), 2) Charlson score, 3) AVF site (radiocephalic, brachiocephalic, basilic), 4) Patency type (primary and assisted), 5) Mortality. This study was approved by the Ethics Committee of the Oswaldo Cruz University Hospital – University of Pernambuco, under registration no. 4.487.269.

**Results:**

Among the 89 patients (age = 70 ± 8, 64% female, 36% frail), 42 (47%) had assisted AVF patency, with 26 (62%) being frail. A positive correlation was found between assisted patency and frailty (p = 0.043). During follow-up (548 to 938 days), 33 (37.1%) patients died, with frail patients showing a higher prevalence of CKD and a higher frequency of assisted patency.

**Conclusion:**

Assisted AVF patency was the most common type among frail elderly patients on hemodialysis. Frailty assessment may be integrated into the clinical evaluation protocol conducted by nurses at HD centers.

## RESUMO

**Objetivo:**

Avaliar a correlação entre a fragilidade e o tipo de patência da FAV em pacientes idosos em hemodiálise (HD).

**Métodos:**

Estudo de coorte retrospectivo com 89 pacientes, de abril de 2019 a junho de 2022. Foram analisados: 1) características dos pacientes segundo a Escala Clínica de Fragilidade (CFS), 2) Índice de Charlson, 3) localização da FAV (radiocefálica, braquiocefálica, basílica), 4) tipo de patência (primária e assistida), 5) mortalidade. O estudo foi aprovado pelo Comitê de Ética do Hospital Universitário Oswaldo Cruz – Universidade de Pernambuco, com registro nº 4.487.269.

**Resultados:**

Dos 89 pacientes (idade = 70 ± 8, 64% mulheres, 36% frágeis), 42 (47%) apresentaram patência assistida da FAV, dos quais 26 (62%) eram frágeis. Encontrou-se correlação positiva entre patência assistida e fragilidade (p = 0,043). Durante o seguimento (548 a 938 dias), 33 pacientes (37,1%) faleceram, e os frágeis apresentaram maior prevalência de DRC e maior frequência de patência assistida.

**Conclusão:**

A patência assistida foi o tipo mais comum entre os pacientes idosos frágeis em HD. A avaliação da fragilidade pode ser integrada ao protocolo clínico de avaliação de enfermagem nos centros de HD.

## INTRODUCTION

The dialysis population is experiencing both growth and aging trends worldwide. Epidemiological data on chronic kidney disease (CKD) in Brazil indicate a prevalence ranging from 10% to 14% in the general population, with rates tripling among individuals over 60 years of age. In primary health care, patients aged 60 and over account for more than 70% of those receiving care^(1)^.

Similarly, among patients undergoing dialysis, the highest prevalence is observed among individuals over 60 years of age. Notably, the age group between 60 and 69 years also exhibits the highest rates of premature mortality attributable to CKD^(1)^. United States Renal Data System statistics indicate that the expected remaining lifetime of an individual aged 75 years and older on hemodialysis (HD) is ≤3.1 years, significantly shorter than the average value of 9.3 years in the general population^(2)^.

In the context of a dialysis-dependent CKD population, predominantly elderly, burdened with multiple comorbidities, and affected by chronic inflammation and treatment-related limitations, the assessment of patient frailty status emerges as a key clinical priority^(3)^.

Frailty is a syndrome characterized by a decline in physical health, making elderly patients more vulnerable to hospitalization and death. Several factors contribute to frailty, including sarcopenia, anemia, uremia, bone fragility, muscle incoordination, and vascular calcification, which are common in HD patients^(3)^.

Early identification of patient frailty by nurses plays a fundamental role in the clinical management of dialysis patients. Regardless of other frailty-related factors, the initiation of dialysis itself tends to exacerbate the degree of frailty due to the elevated inflammatory response associated with this phase of treatment. Nursing assessment of frailty status enables the development of a care plan with targeted interventions aimed at slowing the progression of this condition. Regarding vascular access for hemodialysis (HD), such an evaluation is essential within a multidimensional framework to determine whether the patient is in an optimal condition for referral for arteriovenous fistula (AVF) creation^(4)^.

Guidelines recommend the use of an AVF as the preferred vascular access for HD because of its superior safety profile compared to alternative strategies^(5)^; however, they highlight the importance of individualized assessment for each patient. Therefore, when selecting vascular access, it is crucial to carefully consider the patient’s and life expectancy, particularly among those who are elderly. Factors such as comorbidities, vascular characteristics, frequency of interventions required for maintaining AVF patency and functionality, and associated complications are cited as conditions that reduce the benefits of AVF and must be considered when determining the type of vascular access for HD^(6,7)^.

In frail elderly patients, the benefits of an AVF may be called into question. The concern centers on whether these patients, given their physical and clinical characteristics, can truly derive the expected advantages from an AVF, considering the increased difficulty in maintaining vascular access. Some studies have reported low AVF patency rates in elderly frail patients and have also questioned its utility.

Based on these considerations, this study aimed to evaluate the correlation between frailty and the type of AVF patency in elderly HD patients.

## METHOD

This is a retrospective cohort study that evaluated 89 patients with end-stage kidney disease on HD, from three outpatient dialysis facilities — two in Recife and one in Maceió, Brazil — from April 2019 to June 2022. The inclusion criteria were patients aged 60 years or older, who started thrice-weekly dialysis without AVF.

Dialysis centers were selected based on the availability of Doppler ultrasound equipment and surgical facilities for AVF creation. Additionally, these units followed a standardized protocol for AVF monitoring, from creation to maturation. A non-probabilistic convenience sampling method was used to select the participants.

Medical records were analyzed and the following information was collected: 1) Patients characteristics according to the Clinical Frailty Scale (CFS)^(8)^, 2) Charlson Comorbidity Index^(9)^, 3) AVF site (radiocephalic, brachiocephalic, and basilic), 4) Type of patency (primary and assisted)^(10)^, 5) Mortality.

Data collection was conducted using a standardized questionnaire divided into two sections: [1] records from the patient’s admission to hemodialysis, including analysis of multidisciplinar progress notes, sociodemographic data, etiology of kidney disease, history of comorbidities, and general medical history; and [2] records related to AVF creation and maturation, including referral to the vascular surgeon, AVF surgical creation, first cannulation, and any surgical interventions performed to ensure AVF patency prior to the first use.

For the classification of AVF patency, the criteria proposed by Sidawy et al. were adopted, as follows: [1] Primary patency: AVF functioning for hemodialysis without the need for surgical intervention and/or angioplasty; [2] Assisted primary patency: AVF requiring surgical intervention and/or angioplasty to achieve functionality; [3] No patency: AVF presenting thrombosis immediately after creation and/or before first use. Patient frailty was assessed using the CFS, a 9-point inclusive tool used to summarize an individual’s level of fitness based on clinical evaluation, where higher scores indicate greater frailty and increased clinical risk. The history of comorbidities was used to calculate the Charlson score, according to the validated scale^(9)^. The term “proximal” was used to refer to AVFs located in the arm, whereas “distal” referred to those in the forearm.

Continuous and categorical variables are presented as mean±standard deviation and proportions, respectively, and were compared using Student’s t-test and chi-square test, respectively. Forward stepwise multivariable logistic regression analysis was used to evaluate independent variables. A multivariable Cox regression model accounting for the competing risk of death was also used. Analyses were performed using Stata software v14.2 (StataCorp LP, College Station, Texas, USA).

This study was approved by the Ethics Committee of the Oswaldo Cruz University Hospital – University of Pernambuco, under registration no. 4.487.269.

## RESULTS

The patients analyzed were predominantly female (64%) and had a mean age of 70 ± 8 years. Frailty was observed in 36% of the patients, and 18% were classified as Charlson positive. The most common AVF site was brachiocephalic (46.1%), followed by radiocephalic (33.7%), and basilic (20.2%). Frail patients had a higher prevalence of arm AVFs than non-frail patients (81.3% vs. 57.9%; p = 0.025), as presented in Table 1.

**Table 1:** Age, gender, clinical characteristics, Charlson score, frailty score, AVF site and patency, and death – Recife, PE, Brazil, 2021–2023.

Variables	n = 89
Age, years	70 ± 8
Female, %	64
**CKD etiology**
Hypertension, %	46.1
DM, %	42.7
APKD, %	4.5
Other, %	6.7
**Comorbidities**
DM, %	54
PAD, %	30
HF, %	29
Dementia, %	20
Stroke, %	15
AMI, %	13
Obesity, %	20.2
Charlson positive, %	18
Frailty, %	36
Vein, mm	3.27 ± 0.92
Artery, mm	2.47 ± 0.75
**AVF site**
Radiocephalic, %	33.7
Brachiocephalic, %	46.1
Basilic, %	20.2
Total patency, %	78.6
Assisted patency, %	47.2
No patency	21.4
Death, %	37.1

DM – Diabetes mellitus; APKD – Autosomal Polycystic Kidney Disease; PAD – Peripheral artery disease; HF – Heart failure; AMI – Acute myocardial infarction; AVF – Arteriovenous fistula.

Regarding the Clinical Frailty Scale, 32 (36%) patients were classified as fragile and, among them, 14 (43.7%) presented a Charlson Comorbidity Index > 5 (p <0.001).

Patients with AVF patency (P group, n = 70; age 70 ± 8 years; 35.7% male) and without AVF patency (WP group, n = 19; age 71 ± 7 years; 36.8% male) presented similar characteristics, except for a larger vein diameter in the arm in the P group (3.44 ± 0.87 mm; p < 0.001) and a higher prevalence of upper arm AVF placement (72.9%; p = 0.012), as shown in Table 2.

**Table 2:** Distribution of variables according to AVF patency – Recife, PE, Brazil, 2021–2023.

Variable	With AVF patency (n = 70)	Without AVF patency (n = 19)	p-value
Age, years	70.0 ± 8.0	71.0 ± 8.0	0.45
Male sex, %	35.7	36.8	0.93
Artery, mm	2.57 ± 0.76	2.72 ± 0.71	0.08
Vein, mm	3.44 ± 0.87	2.65 ± 0.88	<0.001
Frailty, %	40	21	0.13
Charlson positive, %	18.6	15.8	0.74
**Comorbidities**			
DM, %	45.7	26.3	0.13
PAD, %	31.4	26.3	0.67
HF, %	28.6	31.5	0.56
AMI, %	14.3	10.5	0.67
Stroke, %	14.3	15.8	0.87
Obesity, %	17.1	31.6	0.16
**AVF site, %**			0.026
Radiocephalic	27.1	57.9
Brachiocephalic	48.6	36.8
Basilic	24.3	5.3
**AVF placement, %**			0.012
Forearm AVF	27.1	57.9
Arm AVF	72.9	42.1

DM – Diabetes mellitus; PAD – Peripheral artery disease; HF – Heart failure; AMI – Acute myocardial infarction; AVF – Arteriovenous fistula.

Considering the type of patency, assisted patency was observed in 42 cases (47%), of which 26 (62%) involved frail patients. A positive correlation was found between assisted patency and frailty (p = 0.043), as shown in Table 3.

**Table 3:** Distribution of variables according to frailty – Recife, PE, Brazil, 2021–2023.

Frailty			
Variables	Frailty (n = 32)	No frailty (n = 57)	p-value
Age, years	72.5 ± 9.16	69.2 ± 6.63	0.03
Vein (mm)	3.42 ± 0.75	3.2 ± 1.00	0.13
Artery (mm)	2.44 ± 0.69	2.49 ± 0.78	0.43
Charlson positive, %	37.5	7	< 0.001
Dementia, %	43.7	7	<0.001
PAD, %	53.1	17.5	<0.001
HF, %	53.1	15.8	<0.001
Stroke, %	34.4	3.5	<0.001
DM, %	56.2	33.3	0.03
**AVF placement, %**			0.025
Forearm, %	18.7	42.1	
Arm, %	81.3	57.9	
Surgical procedure for AVF patency, %	56.2	35.1	0.043
Arm patency, %	43.1	56.9	0.716
Forearm patency, %	31.6	68.4	0.037

PAD – Peripheral artery disease; HF – Heart failure; DM – Diabetes mellitus; AVF – Arteriovenous fistula.

During a follow-up ranging from 548 to 938 days, 33 (37.1%) patients died. Deaths were mostly due to cardiovascular diseases (65.6%), cancer (12.5%), and infections (9.4%). Table 4 shows that patients who died were more likely to be frail and to have a higher prevalence of cardiovascular diseases, and had a higher frequency of assisted patency than those who survived.

**Table 4:** Distribution of variables according to death – Recife, PE, Brazil, 2021–2023.

Death			
Variables	Yes (n = 26)	No (n = 63)	p-value
Age, years	72.92 ± 9.38	69.40 ± 6.77	0.003
Artery, (mm)	2.36 ± 0.51	2.51 ± 0.82	0.63
Vein, (mm)	3.19 ± 0.82	3.31 ± 0.97	0.55
Frailty, %	61.5	25.4	0.001
Charlson positive, %	19.2	17.4	1.0
Dementia, %	34.6	14.3	0.04
PAD, %	38.5	27	0.32
Stroke, %	23.1	11.1	0.19
HF, %	46.2	22.2	0.04
AMI, %	19.2	11.1	0.33
DM, %	46.2	39.7	0.64
Surgical procedure for AVF patency, %	73.1	17.4	0.016
Stenosis, %	69.2	33.3	0.004

PAD – Peripheral artery disease; HF – Heart failure; AMI – Acute myocardial infarction; DM – Diabetes mellitus; AVF – Arteriovenous fistula.

## DISCUSSION

This study showed a positive correlation between frailty and assisted AVF patency in frail patients on HD. It was observed that frail patients required more surgical procedures to maintain AVF patency. This situation may negatively impact the quality of life of elderly frail patients, as it increases hospitalization rates in this population and raises healthcare costs related to vascular access due to the need for repeated interventions, which carry a high likelihood of failure^(11, 12, 13, 14)^.

These findings highlight the critical importance of evaluating HD patients using validated clinical assessment tools related to outcomes such as hospitalization and mortality^(15)^. The use of frailty scales has demonstrated benefits in various medical specialties; however, their application remains limited in nephrology. The literature already underscores the importance of a multidimensional patient assessment, and that nurses—who are central to the management of dialysis care—have a clear responsibility to align the vascular access life plan with the patient’s values, needs, and expectations.

In addition to requiring a greater number of interventions to maintain AVF patency, the present study observed that frail patients underwent more complex surgeries, such as basilic vein superficialization or multiple angioplasties to ensure AVF patency. Such interventions increase the length of hospital stay and are associated with more complications, such as infection and mental disease, conditions that are especially severe in elderly patients. In recent years, frailty has been identified as a negative prognostic factor in surgical patients and has become a stronger predictor of surgical success than age alone in several areas of medicine^(16)^.

Patients undergoing vascular surgery have higher levels of frailty when compared to other surgical patients. This is even more important in HD patients, who commonly present diabetes mellitus and cardiovascular diseases^(17)^. In line with this, the present study found a correlation between the Charlson Comorbidity Index and frailty (p < 0.001). The most common comorbidities were coronary artery disease (CAD), congestive heart failure (CHF), and DM^(14)^. Similarly, Kempe et al. evaluated patients undergoing AVF creation and found a high prevalence of CAD and DM^(11)^.

Several factors influence successful AVF maturation and patency, including arterial vasodilation, arterial and venous outward remodeling, vascular resilience to repeated cannulation, and adequate blood flow free from the interference of tributary veins. These physiological responses are adversely affected by aging, as chronic inflammation and increased thrombogenicity impair both vasodilation and vascular remodeling^(18)^.

The vascular surgeon’s decision to perform a proximal AVF procedure often reflects poor venous quality in the forearm of frail elderly patients. In this context, the shorter life expectancy of these patients, and consequently reduced AVF usage time, may make proximal access a better option. Additionally, forearm AVFs are associated with smaller arterial diameter and lower blood flow, which amplify these challenges. Reinforcing this aspect, our study observed lower AVF patency in frail patients (p = 0.016) when using the forearm veins. Similar findings were reported by Borghese et al., who evaluated 489 patients^(16)^.

While the exact causes of early AVF failure remain unclear, vessel quality is widely recognized as a critical factor. For distal cephalic veins, reduced vein caliber and partial thrombosis have been identified as potential contributors, whereas atherosclerosis and small-caliber radial arteries are significant predictors of failure for arterial access^(19)^.

In the present study, frail patients undergoing proximal AVF showed similar patency rates compared to non-frail patients, but required a greater number of secondary surgical interventions to maintain AFV functionality (p = 0.043). This finding suggests that proximal AVF may be a more viable option in frail elderly patients. These results also reinforce that frail elderly patients with AVF may experience higher hospitalization rates. Furthermore, the present study found that frail elderly patients experienced longer delays before starting AVF use (74.7 ± 64.96 vs. 55.5 ± 33.7; p = 0.07).

Frailty is an important predictor of mortality, as widely documented in the literature^(20,21)^. In this study, a significant association between mortality and frailty was observed (61.5% vs. 25.4%; p < 0.001). Considering both aspects—delayed AVF use and higher mortality—it is essential to evaluate whether AVF creation is indeed the most appropriate strategy for vascular access in frail elderly patients.

Therefore, in frail elderly HD patients, the decision to create an AVF must be based on rigorous clinical criteria. Frailty is associated with a higher number of access-related interventions and increased mortality. These patients are likely to derive fewer short-term benefits from AVF creation due to their reduced life expectancy and overall compromised clinical status. Thus, vascular access decisions must be individualized, taking into account a thorough clinical frailty assessment and the patient’s overall health condition.

This study was retrospective, which is a limitation; however, it revealed that frail elderly HD patients require a careful preoperative evaluation, based on their health circumstances, to decide whether AVF creation is indeed the most appropriate option for vascular access. These patients often have a high mortality risk due to severe cardiovascular diseases and other comorbidities. Further prospective studies must be conducted to validate these correlations.

Early frailty assessment in elderly HD patients by nurses is essential for implementing appropriate care strategies aimed at improving functionality and quality of life. Through this evaluation, nurses can contribute to selecting the most suitable vascular access, considering not only anatomical but also clinical factors. Moreover, active nurse participation in the decision-making process enables more comprehensive and safer planning, facilitating better physical and psychological preparedness, which may result in better clinical outcomes and greater patient satisfaction with their treatment. Therefore, the nurse’s role is pivotal in delivering integrated care and achieving more favorable clinical outcomes, particularly in frail elderly patients.

Developing a structured protocol for vascular access life plan, which considers not only vascular characteristics but also the patient’s life circumstances, preferences, and burden of multicomorbidities, can support safer, more individualized treatment decisions. This strategy reinforces nursing leadership in promoting quality of life and improving clinical outcomes through proactive and holistic care planning.

## CONCLUSION

In elderly frail hemodialysis patients, assisted patency was the most common AVF type. These patients experienced longer waiting times to initiate AFV use, required more complex surgeries and secondary interventions, and had higher hospitalization rates. These findings suggest that these patients may benefit more from alternative types of vascular access.

## Data Availability

All data supporting the findings of this study are available in the Federal University of Pernambuco (UFPE) repository and can be accessed via the following link: https://repositorio.ufpe.br/handle/123456789/55085.
