# Supplementary material for: Frailty and arteriovenous fistula patency in elderly hemodialysis patients: a retrospective cohort study
Source: Rev Esc Enferm USP. 2025 Sep 1;59:e20250121. doi: 10.1590/1980-220X-REEUSP-2025-0121en (PMC12410145; doi:10.1590/1980-220X-REEUSP-2025-0121en)
Supplement: Translation

# Translation: Fragilidad y permeabilidad de la fístula arteriovenosa en pacientes ancianos en hemodiálisis: un estudio de cohortes retrospectivo*

**DOI:** 10.1590/1980-220X-REEUSP-2025-0121es

**Authors:** Polyana Bezerra Mendonça, Rodrigo Bezerra, Flávio Teles, Emmanuelle Tenório Albuquerque Godoi Berenguer de Barros e Silva, Alexandre Cavalcanti de Holanda, Filipe Carrilho de Aguiar, Carolina Sabadoto Brienze, Audes Diógenes Magalhães Feitosa, Wilson Nadruz, Esdras Marques Lins

**Affiliations:** 1 Universidade Federal de Pernambuco, Recife, PE, Brasil.; 2 Universidade Federal de Alagoas, Maceió, AL, Brasil.; 3 Pronto Socorro Cardiológico Universitário de Pernambuco Prof. Luiz Tavares, Recife, PE, Brasil.; 4 Universidade Estadual de Campinas, Campinas, SP, Brasil.

**Keywords:** Fístula Arteriovenosa, Diálisis Renal, Fragilidad, Constricción Patológica, Grado de Desobstrucción Vascular

## Abstract

**Objetivo:**

Evaluar la correlación entre fragilidad y el tipo de permeabilidad de la FAV en pacientes ancianos en HD.

**Métodos:**

Estudio de cohorte retrospectivo con 89 pacientes, desde abril de 2019 hasta junio de 2022. Se analizaron: 1) Características de los pacientes según la Escala de Fragilidad Clínica (CFS), 2) Índice de Charlson, 3) Localización de la FAV (radiocefálica, braquiocefálica, basilical), 4) Tipo de permeabilidad (primaria y asistida), 5) Mortalidad. Este estudio fue aprobado por el Comité de Ética del Hospital Universitario Oswaldo Cruz – Universidade de Pernambuco, con el registro n.º 4.487.269.

**Resultados:**

De los 89 pacientes (edad=70 ± 8, 64% mujeres, 36% frágiles), 42 (47%) presentaron permeabilidad asistida de la FAV, y 26 (62%) eran frágiles. Se encontró una correlación positiva entre la permeabilidad asistida y la fragilidad (p = 0,043). Durante el seguimiento (548 a 938 días), fallecieron 33 (37,1%) pacientes, y los pacientes frágiles mostraron mayor prevalencia de ERC y mayor frecuencia de permeabilidad asistida.

**Conclusión:**

La permeabilidad asistida fue el tipo más común en los pacientes ancianos frágiles en HD. Es posible que la evaluación de la fragilidad se integre en el protocolo de evaluación clínica de enfermeros en los centros de HD.

## INTRODUCCIÓN

La población en diálisis está experimentando tendencias tanto de crecimiento como de envejecimiento en todo el mundo. Los datos epidemiológicos sobre la enfermedad renal crónica (ERC) en Brasil indican una prevalencia que oscila entre el 10% y el 14% en la población general, con tasas que se triplican entre los individuos mayores de 60 años. En la atención primaria, los pacientes mayores de 60 años representan más del 70% de los atendidos^(1)^.

Del mismo modo, entre los pacientes sometidos a diálisis, la mayor prevalencia se observa entre los individuos mayores de 60 años. Cabe destacar que el grupo de edad comprendido entre los 60 y los 69 años también presenta las tasas más elevadas de mortalidad prematura atribuible a la ERC^(1)^. Las estadísticas del Sistema de Datos Renales de los Estados Unidos indican que la esperanza de vida de una persona de 75 años o más en hemodiálisis (HD) es de≤ 3,1 años, significativamente más corta que el valor medio de 9,3 años en la población general^(2)^.

En el contexto de una población con ERC dependiente de diálisis, predominantemente anciana, cargada con múltiples comorbilidades y afectada por inflamación crónica y limitaciones relacionadas con el tratamiento, la evaluación del estado de fragilidad del paciente emerge como una prioridad clínica clave^(3)^.

La fragilidad es un síndrome caracterizado por un deterioro de la salud física, que hace que los pacientes de edad avanzada sean más vulnerables a la hospitalización y a la muerte. Varios factores contribuyen a la fragilidad, incluyendo sarcopenia, anemia, uremia, fragilidad ósea, incoordinación muscular y calcificación vascular, que son comunes en pacientes en HD^(3)^.

La identificación precoz de la fragilidad por parte del personal de enfermería juega un papel fundamental en el manejo clínico de los pacientes en diálisis. Independientemente de otros factores relacionados con la fragilidad, el inicio de la diálisis tiende a exacerbar el grado de fragilidad debido a la elevada respuesta inflamatoria asociada a esta fase del tratamiento. La evaluación de enfermería del estado de fragilidad permite el desarrollo de un plan de cuidados con intervenciones dirigidas a ralentizar la progresión de esta condición. En lo que respecta al acceso vascular para hemodiálisis, dicha evaluación es esencial dentro de un marco multidimensional para determinar si el paciente se encuentra en condiciones óptimas para ser derivado para la creación de una fístula arteriovenosa (FAV)^(4)^.

Las directrices recomiendan el uso de una FAV como acceso vascular preferente para la HD debido a su perfil de seguridad superior en comparación con otras estrategias^(5)^; sin embargo, destacan la importancia de la evaluación individualizada de cada paciente. Por lo tanto, a la hora de seleccionar el acceso vascular, es crucial considerar cuidadosamente la esperanza de vida del paciente, especialmente entre los ancianos. Factores como las comorbilidades, las características vasculares, la frecuencia de las intervenciones necesarias para mantener la permeabilidad y funcionalidad de la FAV y las complicaciones asociadas se citan como condiciones que reducen los beneficios de la FAV y deben tenerse en cuenta a la hora de determinar el tipo de acceso vascular para la HD^(6,7)^.

En los pacientes ancianos frágiles, los beneficios de una FAV pueden ponerse en duda. La preocupación se centra en si estos pacientes, dadas sus características físicas y clínicas, pueden realmente obtener las ventajas esperadas de una FAV, teniendo en cuenta la mayor dificultad para mantener el acceso vascular. Algunos estudios han informado de tasas bajas de permeabilidad de FAV en pacientes ancianos frágiles y también han cuestionado su utilidad.

Basándonos en estas consideraciones, el objetivo de este estudio fue evaluar la correlación entre la fragilidad y el tipo de permeabilidad de las FAV en pacientes ancianos en HD.

## MÉTODO

Se trata de un estudio de cohortes retrospectivo que evaluó 89 pacientes con insuficiencia renal terminal en HD, de tres centros de diálisis ambulatoria - dos en Recife y uno en Maceió, Brasil - desde abril de 2019 hasta junio de 2022. Los criterios de inclusión fueron pacientes de 60 años o más, que iniciaron diálisis tres veces por semana sin FAV.

Los centros de diálisis se seleccionaron en función de la disponibilidad de equipos de ecografía Doppler e instalaciones quirúrgicas para la creación de FAV. Además, estas unidades siguieron un protocolo estandarizado para la monitorización de las FAV, desde la creación hasta la maduración. Para seleccionar a los participantes se utilizó un método de muestreo de conveniencia no probabilístico.

Se analizaron las historias clínicas y se recogió la siguiente información: 1) Características de los pacientes según la escala de fragilidad clínica (Clinical Frailty Scale - CFS)^(8)^, 2) Índice de comorbilidad de Charlson^(9)^, 3) Localización de la FAV (radiocefálica, braquiocefálica y basílica), 4) Tipo de permeabilidad (primaria y asistida)^(10)^, 5) Mortalidad.

La recolección de datos se realizó mediante un cuestionario estandarizado dividido en dos secciones: [1] registros del ingreso del paciente en hemodiálisis, incluido el análisis de anotaciones clínicas de evolución multidisciplinares, datos sociodemográficos, etiología de la enfermedad renal, antecedentes de comorbilidades y antecedentes médicos generales; y 2] registros relacionados con la creación y maduración de la FAV, incluida la derivación al cirujano vascular, la creación quirúrgica de la FAV, la primera canulación y cualquier intervención quirúrgica realizada para garantizar la permeabilidad de la FAV antes del primer uso.

Para la clasificación de la permeabilidad de las FAV se adoptaron los criterios propuestos por Sidawy et al.^(10)^: [1] Permeabilidad primaria: FAV funcional para hemodiálisis sin necesidad de intervención quirúrgica y/o angioplastia; [2] permeabilidad primaria asistida: FAV que requiere intervención quirúrgica y/o angioplastia para alcanzar la funcionalidad; [3] No permeable: FAV que presenta trombosis inmediatamente después de su creación y/o antes de su primer uso. La fragilidad de los pacientes se evaluó mediante el CFS, una herramienta inclusiva de 9 puntos utilizada para resumir el nivel de aptitud física de un individuo en función de la evaluación clínica, donde las puntuaciones más altas indican mayor fragilidad y mayor riesgo clínico. El historial de comorbilidades se utilizó para calcular la puntuación de Charlson, según la escala validada^(9)^. El término “proximal” se utilizó para referirse a las FAV localizadas en el brazo, mientras que “distal” se refería a las del antebrazo.

Las variables continuas y categóricas se presentan como media ± desviación estándar y proporciones, respectivamente, y se compararon mediante la prueba t de Student y la prueba chi-cuadrado, respectivamente. Para evaluar las variables independientes se utilizó un análisis de regresión logística multivariable por pasos hacia delante. También se utilizó un modelo de regresión de Cox multivariable que tenía en cuenta el riesgo competitivo de muerte. Los análisis se realizaron con el programa Stata v14,2 (StataCorp LP, College Station, Texas, EE.UU.).

Este estudio fue aprobado por el Comité de Ética del Hospital Universitario Oswaldo Cruz - Universidad de Pernambuco, bajo el registro no. 4.487.269.

## RESULTADOS

Los pacientes analizados eran predominantemente mujeres (64%) y tenían una edad media de 70 ± 8 años. Se observó fragilidad en 36% de los pacientes, y 18% fueron clasificados como Charlson positivo. La localización más frecuente de las FAV fue la braquiocefálica (46,1%), seguida de la radiocefálica (33,7%) y la basílica (20,2%). Los pacientes frágiles tenían una mayor prevalencia de FAV en el brazo que los no frágiles (81,3% frente a 57,9%; p = 0,025), como se muestra en la Tabla 1.

**Tabla 1:** Edad, sexo, características clínicas, puntuación de Charlson, puntuación de fragilidad, localización y permeabilidad de la FAV y muerte – Recife, PE, Brasil, 2021–2023.

Variables	n = 89
Edad, años	70 ± 8
Sexo femenino, %.	64
**Etiología de la ERC**	
Hipertensión arterial, %.	46,1
DM, %.	42,7
APKD, %	4,5
Otros, %	6,7
**Comorbilidades**	
DM (%)	54
EAP, %	30
INSUFICIENCIA CARDIACA, %.	29
Demencia	20
Ictus,	15
IAM, %	13
Obesidad (%)	20,2
Charlson positivo, %	18
Fragilidad (%)	36
Vena, mm	3,27 ± 0,92
Arteria, mm	2,47 ± 0,75
**Localización de la FAV**	
Radiocefálica, %.	33,7
Braquiocefálica, %.	46,1
Basílica, %.	20,2
Permeabilidad total, %	78,6
Permeabilidad asistida, %.	47,2
No permeabilidad	21,4
Fallecimiento, %.	37,1

DM: diabetes mellitus; APKD: poliquistosis renal autosómica dominante; EAP: enfermedad de arteriopatía periférica; IC: insuficiencia cardiaca; IAM: infarto agudo de miocardio; FAV: fístula arteriovenosa.

En cuanto a la Escala de Fragilidad Clínica, 32 (36%) pacientes fueron clasificados como frágiles y, entre ellos, 14 (43,7%) presentaban un Índice de Comorbilidad de Charlson > 5 (p < 0,001).

Los pacientes con permeabilidad de FAV (grupo P, n = 70; edad 70 ± 8 años; 35,7% varones) y sin permeabilidad de FAV (grupo WP, n = 19; edad 71 ± 7 años; 36,8% varones) presentaban características similares, excepto por un mayor diámetro venoso en el brazo en el grupo P (3,44 ± 0,87 mm; p < 0,001) y una mayor prevalencia de colocación de FAV en la parte superior del brazo (72,9%; p = 0,012), como se muestra en la Tabla 2.

**Tabla 2:** Distribución de las variables de acuerdo con la permeabilidad de las FAV – Recife, PE, Brasil, 2021–2023.

Variable	Con permeabilidad de FAV (n = 70)	Sin permeabilidad de FAV (n = 19)	p-valor
Edad, años	70,0 ± 8,0	71,0 ± 8,0	0,45
Sexo masculino (%)	35,7	36,8	0,93
Arteria, mm	2,57 ± 0,76	2,72 ± 0,71	0,08
Vena, mm	3,44 ± 0,87	2,65 ± 0,88	<0,001
Fragilidad (%)	40	21	0,13
Charlson positivo, %	18,6	15,8	0,74
**Comorbilidades**			
DM, %	45,7	26,3	0,13
EAP (%)	31,4	26,3	0,67
IC, %	28,6	31,5	0,56
IAM, %	14,3	10,5	0,67
Ictus (%)	14,3	15,8	0,87
Obesidad (%)	17,1	31,6	0,16
**Localización de la FAV, %.**			0,026
Radiocefálica	27,1	57,9
Braquiocefálico	48,6	36,8
Basílica	24,3	5,3
**Colocación de FAV, %.**			0,012
FAV antebrazo	27,1	57,9
FAV brazo	72,9	42,1

DM: diabetes mellitus; EAP: enfermedad de arteriopatía periférica; IC: insuficiencia cardiaca; IAM: infarto agudo de miocardio; FAV: fístula arteriovenosa.

Teniendo en cuenta el tipo de permeabilidad, se observó permeabilidad asistida en 42 casos (47%), de los cuales 26 (62%) correspondían a pacientes frágiles. Se encontró una correlación positiva entre permeabilidad asistida y fragilidad (p = 0,043), como se muestra en la Tabla 3.

**Tabla 3:** Distribución de variables según fragilidad – Recife, PE, Brasil, 2021–2023.

Fragilidad			
Variables	Fragilidad (n = 32)	No fragilidad (n = 57)	p-valor
Edad, años	72,5 ± 9,16	69,2 ± 6,63	0,03
Vena (mm)	3,42 ± 0,75	3,2 ± 1,00	0,13
Arteria (mm)	2,44 ± 0,69	2,49 ± 0,78	0,43
Charlson positivo, %	37,5	7	< 0,001
Demencia, %	43,7	7	<0,001
EAP, %	53,1	17,5	<0,001
IC, %	53,1	15,8	<0,001
Ictus, %.	34,4	3,5	<0,001
DM, %	56,2	33,3	0,03
**Colocación de FAV, %.**			0,025
Antebrazo (%)	18,7	42,1	
Brazo (%)	81,3	57,9	
Procedimiento quirúrgico para la permeabilidad de la FAV, %.	56,2	35,1	0,043
Permeabilidad del brazo, %.	43,1	56,9	0,716
Permeabilidad del antebrazo, en %.	31,6	68,4	0,037

EAP: enfermedad de arteriopatía periférica; IC: insuficiencia cardiaca; DM: diabetes mellitus; FAV: fístula arteriovenosa.

Durante un seguimiento que osciló entre 548 y 938 días, fallecieron 33 (37,1%) pacientes. Las muertes se debieron principalmente a enfermedades cardiovasculares (65,6%), cáncer (12,5%) e infecciones (9,4%). La Tabla 4 muestra que los pacientes que fallecieron eran más propensos a ser frágiles y a presentar una mayor prevalencia de enfermedades cardiovasculares, y tenían una mayor frecuencia de permeabilidad asistida que los que sobrevivieron.

**Tabla 4:** Distribución de las variables según el fallecimiento – Recife, PE, Brasil, 2021–2023.

Fallecimiento			
Variables	Sí (n = 26)	No (n = 63)	p-valor
Edad, años	72,92 ± 9,38	69,40 ± 6,77	0,003
Arteria, (mm)	2,36 ± 0,51	2,51 ± 0,82	0,63
Vena, (mm)	3,19 ± 0,82	3,31 ± 0,97	0,55
Fragilidad %	61,5	25,4	0,001
Charlson positivo, %	19,2	17,4	1,0
Demencia %	34,6	14,3	0,04
EAP, %.	38,5	27	0,32
Ictus %	23,1	11,1	0,19
IC, %.	46,2	22,2	0,04
IAM, %	19,2	11,1	0,33
DM %	46,2	39,7	0,64
Procedimiento quirúrgico para la permeabilidad de la FAV, %.	73,1	17,4	0,016
Estenosis (%)	69,2	33,3	0,004

EAP: arteriopatía periférica; IC: insuficiencia cardiaca; IAM: infarto agudo de miocardio; DM: diabetes mellitus; FAV: fístula arteriovenosa.

## DISCUSIÓN

Este estudio mostró una correlación positiva entre fragilidad y permeabilidad de FAV asistida en pacientes frágiles en HD. Se observó que los pacientes frágiles requerían más procedimientos quirúrgicos para mantener la permeabilidad de la FAV. Esta situación puede repercutir negativamente en la calidad de vida de los pacientes frágiles de edad avanzada, ya que aumenta las tasas de hospitalización en esta población y eleva los costes sanitarios relacionados con el acceso vascular debido a la necesidad de intervenciones repetidas, que conllevan una alta probabilidad de fracaso^(11, 12, 13, 14)^.

Estos hallazgos ponen de manifiesto la importancia crítica de evaluar a los pacientes en HD utilizando herramientas validadas de evaluación clínica relacionadas con resultados como la hospitalización y la mortalidad^(15)^. El uso de escalas de fragilidad ha demostrado beneficios en varias especialidades médicas; sin embargo, su aplicación sigue siendo limitada en nefrología. La literatura ya subraya la importancia de una evaluación multidimensional del paciente, y que las enfermeras -que son fundamentales en la gestión de los cuidados de diálisis- tienen la clara responsabilidad de alinear el plan de vida del acceso vascular con los valores, necesidades y expectativas del paciente.

Además de requerir un mayor número de intervenciones para mantener la permeabilidad de la FAV, el presente estudio observó que los pacientes frágiles se sometían a cirugías más complejas, como la superficialización de la vena basílica o angioplastias múltiples para asegurar la permeabilidad de la FAV. Tales intervenciones aumentan la duración de la estancia hospitalaria y se asocian a más complicaciones, como infección y problemas de salud mental, condiciones que son especialmente graves en los pacientes ancianos. En los últimos años, la fragilidad se ha identificado como un factor pronóstico negativo en pacientes quirúrgicos y se ha convertido en un factor predictivo del éxito quirúrgico más importante que la edad por sí sola en varias áreas de la medicina^(16)^.

Los pacientes sometidos a cirugía vascular presentan mayores niveles de fragilidad en comparación con otros pacientes quirúrgicos. Esto es aún más importante en los pacientes en HD, que comúnmente presentan diabetes mellitus y enfermedades cardiovasculares^(17)^. En línea con esto, el presente estudio encontró una correlación entre el Índice de Comorbilidad de Charlson y la fragilidad (p < 0,001). Las comorbilidades más comunes fueron la enfermedad arterial coronaria (EAC), la insuficiencia cardiaca congestiva (ICC) y la DM^(14)^. De forma similar, Kempe et al. evaluaron a pacientes sometidos a creación de FAV y hallaron una alta prevalencia de EAC y DM^(11)^.

Varios factores influyen en la maduración y permeabilidad satisfactorias de las FAV, como la vasodilatación arterial, el remodelado arterial y venoso, la resistencia vascular a la canulación repetida y un flujo sanguíneo adecuado libre de la interferencia de las venas tributarias. Estas respuestas fisiológicas se ven afectadas negativamente por el envejecimiento, ya que la inflamación crónica y el aumento de la trombogenicidad deterioran tanto la vasodilatación como la remodelación vascular^(18)^.

La decisión del cirujano vascular de realizar una intervención de FAV proximal refleja a menudo la mala calidad venosa en el antebrazo de los pacientes ancianos frágiles. En este contexto, la menor esperanza de vida de estos pacientes y, en consecuencia, el menor tiempo de uso de la FAV, pueden hacer que el acceso proximal sea una mejor opción. Además, las FAV de antebrazo se asocian a un diámetro arterial menor y a un flujo sanguíneo más bajo, lo que amplifica estas dificultades. Reforzando este aspecto, nuestro estudio observó una menor permeabilidad de las FAV en pacientes frágiles (p = 0,016) cuando se utilizaban las venas del antebrazo. Borghese et al, que evaluaron a 489 pacientes^(16)^ comunicaron resultados similares.

Aunque las causas exactas del fracaso precoz de las FAV siguen sin estar claras, la calidad de los vasos está ampliamente reconocida como un factor crítico. En el caso de las venas cefálicas distales, el calibre reducido de la vena y la trombosis parcial se han identificado como posibles factores contribuyentes, mientras que la aterosclerosis y las arterias radiales de pequeño calibre son predictores significativos del fracaso del acceso arterial^(19)^.

En el presente estudio, los pacientes frágiles sometidos a FAV proximal mostraron tasas de permeabilidad similares en comparación con los pacientes no frágiles, pero precisaron un mayor número de intervenciones quirúrgicas secundarias para mantener la funcionalidad de la FAV (p = 0,043). Este hallazgo sugiere que la FAV proximal puede ser una opción más viable en pacientes ancianos frágiles. Estos resultados también refuerzan la idea de que los pacientes ancianos frágiles con FAV pueden experimentar mayores tasas de hospitalización. Además, en el presente estudio se observó que los pacientes ancianos frágiles experimentaban mayores retrasos antes de iniciar el uso de la FAV (74,7 ± 64,96 frente a 55,5 ± 33,7; p = 0,07).

La fragilidad es un importante factor predictivo de mortalidad, como se ha documentado ampliamente en la literatura^(20,21)^. En este estudio, se observó una asociación significativa entre mortalidad y fragilidad (61,5% vs. 25,4%; p < 0,001). Teniendo en cuenta ambos aspectos -retraso en el uso de FAV y mayor mortalidad- es esencial evaluar si la creación de FAV es realmente la estrategia más adecuada para el acceso vascular en pacientes ancianos frágiles.

Por lo tanto, en los pacientes ancianos frágiles en HD, la decisión de crear una FAV debe basarse en criterios clínicos rigurosos. La fragilidad se asocia a un mayor número de intervenciones relacionadas con el acceso y a una mayor mortalidad. Es probable que estos pacientes obtengan menos beneficios a corto plazo de la creación de una FAV debido a su reducida esperanza de vida y a su estado clínico general comprometido. Así pues, las decisiones sobre el acceso vascular deben ser individualizadas, teniendo en cuenta una evaluación clínica exhaustiva de la fragilidad y el estado de salud general del paciente.

Este estudio fue retrospectivo, lo que supone una limitación; sin embargo, reveló que los pacientes ancianos frágiles en HD requieren una evaluación preoperatoria cuidadosa, basada en sus circunstancias de salud, para decidir si la creación de FAV es realmente la opción más adecuada para el acceso vascular. Estos pacientes suelen tener un alto riesgo de mortalidad debido a enfermedades cardiovasculares graves y otras comorbilidades. Deben realizarse más estudios prospectivos para validar estas correlaciones.

La evaluación precoz de la fragilidad en pacientes ancianos en HD por parte del personal de enfermería es esencial para implementar estrategias de cuidados adecuadas dirigidas a mejorar la funcionalidad y la calidad de vida. A través de esta evaluación, las enfermeras pueden contribuir a seleccionar el acceso vascular más adecuado, teniendo en cuenta no sólo factores anatómicos sino también clínicos. Además, la participación activa de la enfermera en el proceso de toma de decisiones permite una planificación más completa y segura, facilitando una mejor preparación física y psicológica, lo que puede dar lugar a mejores resultados clínicos y a una mayor satisfacción del paciente con su tratamiento. Por lo tanto, el papel de la enfermera es fundamental para prestar una atención integrada y lograr resultados clínicos más favorables, especialmente en pacientes ancianos frágiles.

El desarrollo de un protocolo estructurado para el plan de vida del acceso vascular, que tenga en cuenta no sólo las características vasculares sino también las circunstancias vitales del paciente, sus preferencias y la carga de multi-comorbilidades, puede respaldar la toma de decisiones terapéuticas más seguras e individualizadas. Esta estrategia refuerza el liderazgo de la enfermería en la promoción de la calidad de vida y la mejora de los resultados clínicos mediante una planificación de los cuidados proactiva y holística.

## CONCLUSIÓN

En los pacientes ancianos frágiles en hemodiálisis, la permeabilidad asistida fue el tipo de FAV más frecuente. Estos pacientes experimentaron tiempos de espera más prolongados para iniciar el uso de FAV, requirieron cirugías e intervenciones secundarias más complejas y presentaron tasas de hospitalización más elevadas. Estos hallazgos sugieren que estos pacientes pueden beneficiarse más de tipos alternativos de acceso vascular.

## Data Availability

Todos los datos que respaldan los hallazgos de este estudio están disponibles en el repositorio de la Universidad Federal de Pernambuco (UFPE) y se puede acceder a ellos a través del siguiente enlace: https://repositorio.ufpe.br/handle/123456789/55085.
